# Genetic Disorders of the Extracellular Matrix: From Cell and Gene Therapy to Future Applications in Regenerative Medicine

**DOI:** 10.1146/annurev-genom-083117-021702

**Published:** 2022-05-10

**Authors:** Shukti Chakravarti, Elena Enzo, Maithê Rocha Monteiro de Barros, Maria Benedetta Rizzarda Maffezzoni, Graziella Pellegrini

**Affiliations:** 1 Department of Ophthalmology and Department of Pathology, Grossman School of Medicine, New York University, New York, NY, USA; 2 Center for Regenerative Medicine “Stefano Ferrari,” University of Modena and Reggio Emilia, Modena, Italy

**Keywords:** laminins, perlecan, collagens, Alport syndrome, epidermolysis bullosa, chondrodysplasia

## Abstract

Metazoans have evolved to produce various types of extracellular matrix (ECM) that provide structural support, cell adhesion, cell–cell communication, and regulated exposure to external cues. Epithelial cells produce and adhere to a specialized sheet-like ECM, the basement membrane, that is critical for cellular homeostasis and tissue integrity. Mesenchymal cells, such as chondrocytes in cartilaginous tissues and keratocytes in the corneal stroma, produce a pericellular matrix that presents optimal levels of growth factors, cytokines, chemokines, and nutrients to the cell and regulates mechanosensory signals through specific cytoskeletal and cell surface receptor interactions. Here, we discuss laminins, collagen types IV and VII, and perlecan, which are major components of these two types of ECM. We examine genetic defects in these components that cause basement membrane pathologies such as epidermolysis bullosa, Alport syndrome, rare pericellular matrix–related chondrodysplasias, and corneal keratoconus and discuss recent advances in cell and gene therapies being developed for some of these disorders.

## INTRODUCTION

Produced by all metazoans, the extracellular matrix (ECM) is a dynamic extracellular collection of interacting glycoproteins, proteoglycans, and glycosaminoglycans (42, 129). Among its abundant members are laminins, collagens, perlecan, and nidogens. Initially secreted in the extracellular space, these macromolecules become organized into cell scaffolds that provide structural support, a protective barrier, and a means for regulated communication between cells (72, 164). Growth factors, morphogens, proteinases, regulatory macromolecules, and serum are incorporated into this matrix, creating a complex mechanotransduction platform for correct homeostatic responses to internal and external stimuli (122).

Mutations in ECM-encoding genes that cause severe diseases have led to fundamental insights into functions of the ECM in the epithelia (60) and mesenchymal connective tissues (49, 150). A comprehensive summary of ECM-associated diseases can be found in Table 1. Although the majority of the genes listed in the table are ECM encoding, we have included a few that are not because of their implications in ECM pathologies. An understanding of genetic defects of the ECM is slowly beginning to shape regenerative medicine, which combines tissue engineering, prostheses, and scaffolds with cell and gene therapy to restore a functional ECM and improve patient survival. In this review, we focus on four genetic disorders that lead to pathologies of the basement membrane (BM) and pericellular matrix (PCM): epidermolysis bullosa (EB); Alport syndrome (ATS); and two chondrodysplasias, Schwartz–Jampel syndrome type 1 (SJS1) and dyssegmental dysplasia, Silverman–Handmaker type (DDSH). We examine existing therapeutic approaches and compare their advantages and disadvantages as a paradigm for future therapeutic options for ECM-related genetic diseases.

The epithelia and cartilage have some of the most successful examples of tissue regeneration in vitro. Laminins, collagens, and perlecan form a specialized ECM, called the basal lamina or BM in epithelia and the PCM in chondrocytes. Green (51) pioneered the use of epithelial cells in autologous skin reconstitution for burn patients in the 1980s. Limbal stem cells have since been used in cell therapy to cure unilateral or partial bilateral limbal stem cell deficiency in the corneal epithelium using an autologous ex vivo regeneration approach (123). Holoclar, the first stem cell–based drug, was approved for commercialization as an advanced therapy medicinal product (ATMP) in Europe in 2015. Genetically corrected epidermal keratinocytes have been successfully applied to overcome the effects of *LAMB3* mutations in junctional epidermolysis bullosa (JEB), a devastating skin disease (100). Autologous chondrocyte implantation is another ATMP that relies on ex vivo amplification of chondrocytes in matrix-associated spheroids and implantation of these spheroids at sites of cartilage damage (154). Another advancement on this front is autologous chondrocyte sheets for cartilage defects of the knee (130). Combining this type of ex vivo autologous cell culturing with somatic gene therapy is proving to be promising. However, no cell-based treatments have been developed for the severe chondrodysplasias caused by mutations in the *HSPG2* gene, which encodes perlecan. For ATS, bone marrow transplantation tested in the *Col4a3* mutant mouse model shows incorporation of a normal α3 chain and considerable phenotype rescue in the recipient mice (90). Additional pharmaceutical approaches that are beyond the scope of the current review include delaying end-stage kidney disease by using pharmacological agents such as antihypertensive renin-angiotensin-aldosterone-system inhibition to reduce glomerular capillary pressure in ATS (81).

Finally, we discuss recent efforts in permanent or transient correction of genetic defects. One approach introduces an antisense oligonucleotide (AON) that hybridizes to specific exons, resulting in targeted in-frame splicing out in pre-mRNA to yield partially functional proteins (58, 149). CRISPR-Cas9-mediated gene editing is another powerful strategy that is being actively pursued for treatments of JEB (9) and dystrophic epidermolysis bullosa (DEB) (35, 85, 119).

## THE BASEMENT MEMBRANE

First described by Bowman & Todd (12) in 1840, the BM is a sheet-like cell-adherent ECM produced by epithelial, endothelial, and muscle cells and adipocytes. It serves as an extension of the plasma membrane and cytoskeleton and provides biomechanical support and a signaling interface between the cell and its environment to mediate cell growth, differentiation, remodeling, and repair (6, 57, 122). It is usually 50–100 nm thick, but much thicker BMs exist, as in the lens capsule, the renal globular BM, the mouse and rat parietal yolk sac BM or Reichert’s membrane, and the corneal Descemet’s membrane (29, 33, 164). It is also dynamic, increasing in thickness during development and aging and in various pathologies (57).

**Figure 1:**
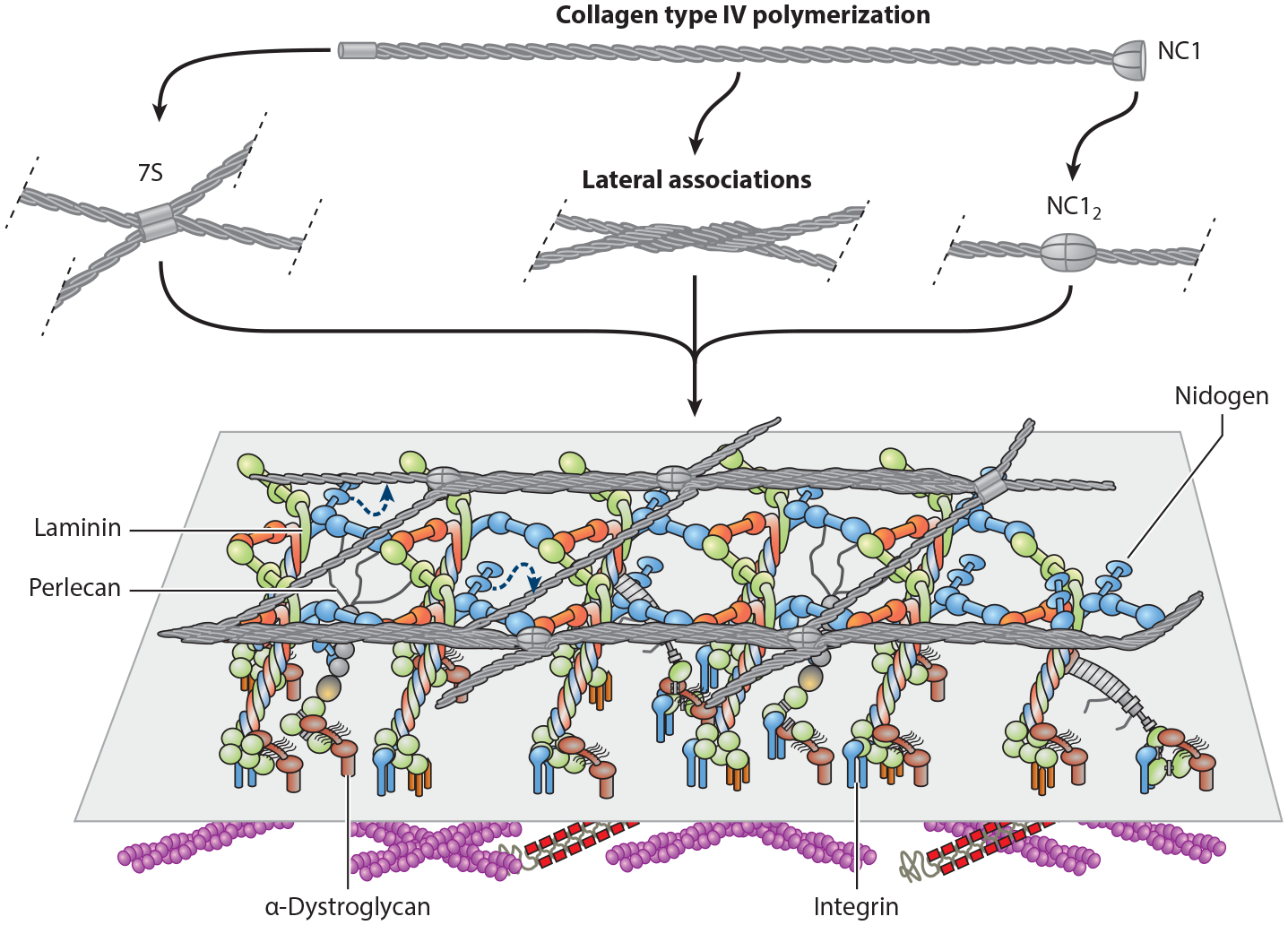
Basement membrane assembly. Laminin self-assembles into a polymer and binds to integrins and α-dystroglycan associated with the plasma membrane. Collagen type IV trimers form a tight network through interactions at the 7S and noncollagenous 1 (NC1) domains and lateral associations of the chains. The collagen type IV polymer is bridged to the laminin polymer by nidogen and the heparan sulfate side chains of perlecan. Figure adapted with permission from Reference 164.

An understanding of the molecular nature of BMs first came in the 1970s with biochemical studies of the Engelbreth–Holm–Swarm sarcoma BM extracts also known as Matrigel (83, 118); purification of laminins (23, 147), *HSPG2*/perlecan (63), and entactin/nidogen 1 (23, 37, 146); and molecular cloning of the corresponding genes. Additional minor components include fibronectin, netrins, usherin, agrin, and other proteins identified by proteomic approaches (109). BM assembly is considered to occur through a multistep process of laminin self-assembly (reviewed in 164) and anchoring of the polymer to the cell surface, which we review in the section titled The Laminin Family (Figure 1). BM–cell adhesion occurs through interactions of laminins with integrins, α-dystroglycan, heparan sulfates, and sulfated glycolipids (69, 101). Collagen type IV, the other major component of the BM, also self-assembles to form a network, which is bridged to the laminin polymer by nidogens and heparan sulfate side chains of perlecan (122, 145). The laminin polymer is integral to BM assembly and embryonic development, as its absence in *Lamc1*-null mice leads to the lack of a BM and preimplantation lethality (140). Perlecan is another major component of BMs that we discuss later in the context of chondrodysplasias (see the section titled Perlecan and the section titled Chondrodysplasias: Schwartz–Jampel Syndrome Type 1 and Dyssegmental Dysplasia, Silverman–Handmaker Type) (115).

## THE PERICELLULAR MATRIX

**Figure 2:**
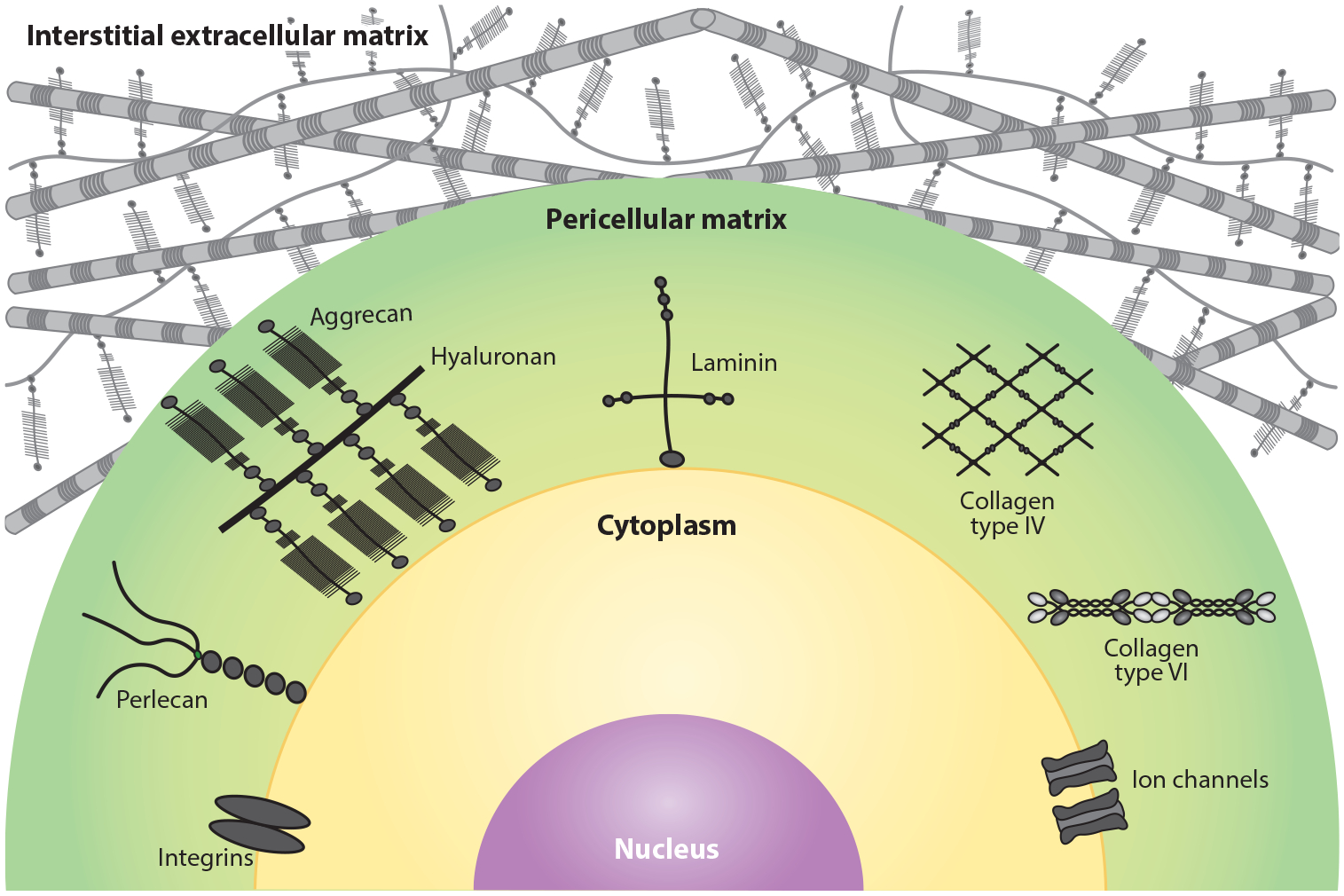
Pericellular matrix surrounding a cell, featuring integrins, perlecan, an aggrecan and hyaluronan complex, laminin, collagen types IV and VI, and ion channels. The pericellular matrix is embedded in the interstitial extracellular matrix.

The PCM is a 2–4-μm-thick zone of matrix that surrounds mesenchymal cells and connects them to the deeper interstitial ECM (18) (Figure 2). It contains the major BM proteins—laminins, perlecan, collagen type IV, and nidogen—which prompted one study to propose that it is functionally equivalent to the BM (88). However, the PCM is also rich in interstitial ECM components, collagen type VI, aggrecan, and hyaluronan networks. Perlecan has a prominent role in the PCM; its interactions with collagen types VI (64) and XI (138), cell-adhesive proteins, and the small leucinerich-repeat proteoglycans decorin and biglycan stabilize the PCM around chondrocytes (159). Decorin may help to retain aggrecan in the PCM, and *Dcn*-null mouse cartilage displays biomechanical weakness (21). Lumican, another proteoglycan similar to decorin, associates with the cell surface and stabilizes cell surface lipid rafts that may further mediate cell–PCM crosstalk (96). Overall, the PCM serves multiple purposes, from maintaining homeostatic levels of osmolarity, growth factors, cytokines, and antimicrobial barriers to mediating mechanosensation and cellular metabolism (55). In addition, perlecan and the other proteoglycans of the PCM help to retain water and regulate Na^+^/K^+^ gradients and intracellular signaling (55). Historically, the PCM has been described for osteocytes (76) and chondrocytes (54) embedded in a dense connective tissue, but the PCM applies broadly to mesenchymal cells in general. For example, keratocytes, a type of fibroblast in the corneal stroma, are also embedded in a PCM (62), and perturbations in keratocyte–PCM interactions may be important in a degenerative disease of the cornea called keratoconus.

### The Laminin Family

The laminins are large (400–800 kDa) heterotrimeric molecules of α, β, and γ chains that assemble from the C-terminal end to form a long coiled-coil domain that makes up the long arm of the heterotrimer (71, 95). Mammals have 12 genes that encode five α, four β, and three γ polypeptides. Of the >60 possible combinations, only 16 αβγ trimers have been identified in vivo and named according to their subunit composition. These isoforms show development- and tissue-specific expression and harbor multiple functions, which include the stem cell niche and cues for epithelial and endothelial cell and cardiomyocyte differentiation (161).

The classical laminin (Lm111) was characterized from Matrigel (83), and this isoform is widely expressed during embryogenesis (117, 161). The Lm511 and Lm521 isoforms are ubiquitous in adult tissues, Lm211 in the muscle BM (36, 153), and Lm411 and Lm421 in the vascular endothelial BM (163). Lm332 is present in the subepithelial BM of the skin and is a major regulator of the epidermal–dermal junction (19, 78). Mutations in *LAMA3*, *LAMB3*, and *LAMC2*, encoding the Lm332 isoform, cause the EB types (61) that are reviewed here. The α1 and α5 chains are longer than the other chains, and isomers with these have a cross shape. The three short arms comprise the N-terminal ends of α1 or α5, β, and γ chains, with a terminal globular domain, and one (β1–2 or γ1–3) or two (α1, α2, and/or α5) other internal globular domains (reviewed in 161). Interactions between the short-arm terminal globular domains allow the polymerization of Lm332 into the BM (69). At the C-terminal coiled-coil tail, the α chain extends into five globular domains (LG1–5), of which LG1–3 bind integrin receptors α3β1, α6β1, and α6β4, respectively, while LG4 and LG5 bind to dystroglycan and the heparan sulfate side chains of proteoglycans, respectively, to mediate cell adhesion and signaling (6, 68). Mutations that disrupt the Lm332 isoform or its integrin receptors cause mild to severe JEB, as discussed in the section titled Epidermolysis Bullosa.

### The Collagen Family

Collagens are the most abundant proteins in the body, with 28 mammalian collagen types encoded by at least 45 distinct genes (for reviews, see 50, 103, 126). All collagens have a contiguous or interrupted triple-helical domain with a tripeptide repeat of Gly-Xaa-Yaa, where Xaa and Yaa are frequently proline and hydroxyproline, respectively. The collagenous domain is flanked at the N and C termini by noncollagenous propeptides. Three parallel procollagen chains begin folding at their C-terminal ends in the endoplasmic reticulum, and fully folded trimers are exported and assembled into supramolecular structures once their propeptides are cleaved. Thus, genetic defects in accessory enzymes or any one collagen chain (for heterotrimeric types) can affect assembly and secretion of the functional trimeric protein. Several reviews are available on collagen-modifying enzymes, collagen assembly, mechanisms of quality control of procollagens in cells, and export mechanisms (15, 50, 73, 103, 125, 126).

Collagen type IV is a major BM component (Figure 1) that we examine here and discuss in the context of ATS in the section titled Alport Syndrome. Collagen types XIV, XV, XVIII, XIX, XXI, and XXII are minor components and are not discussed further. Collagen type IV tethers cells to the BM through interactions with the integrin receptors α1β1, α2β1, and αvβ3 and with discoidin domain receptors (39, 45, 82). It is a heterotrimer or protomer of three different α chains encoded by six homologous genes arranged in a unique pairwise head-to-head organization: *COL4A1* and *COL4A2* on chromosome 13, *COL4A3* and *COL4A4* on chromosome 2, and *COL4A5* and *COL4A6* on the X chromosome (167). Of all possible combinations, only 16 collagen type IV heterotrimers are known to exist in nature due to their regulated expression and chain-specific interactions (82). The N-terminal end of each α chain associates to form a proteolytically resistant fragment, termed 7S because of the 7S sedimentation coefficient of this complex; a collagenous central domain; and a C-terminal noncollagenous 1 (NC1) domain (Figure 1). Heterotrimer assembly begins intracellularly through chain-specific interactions of the NC1 domains (91). Extracellular supramolecular assembly was initially proposed to occur through the binding of four protomers at the 7S domains and end-to-end joining of two protomers (15, 148, 165). Subsequent structural studies of amniotic BM and in vitro collagen type IV polymers indicated an additional noncovalent lateral association between chains to yield a tighter meshwork that is regulated further by the local milieu and the plasma membrane (165).

Two other collagens, types VII and XVII, are included in this review for their significant roles in DEB and JEB, respectively (48, 126). Collagen type VII, a homotrimer, encoded by *COL7A1*, forms anchoring fibrils (~440 nm) at BM-interstitial ECM junctions. Two collagen type VII molecules associate at their C-terminal ends to form a U-shaped duplex, while their free NC1 domains bind laminin and collagen type IV in BMs (14). This sling-like structure traps interstitial collagen fibrils and macromolecules to tether the epithelial BM to the connective tissue underneath (133). The plasma membrane–bound collagen type XVII, also known as the 180-kDa bullous pemphigoid antigen (BP180), forms hemidesmosomal and cell–cell junctions in basal keratinocytes (reviewed in 113). It is an α1 homotrimer with a cytoplasmic N-terminal globular domain, a 23-amino-acid-long transmembrane, and an extracellular interrupted collagenous domain (47, 93). Collagen type XVII stabilizes epidermal–dermal junctions by binding laminin (Lm332) and possibly collagen type IV at its C terminus and by binding hemidesmosomal BP230, plectin, and β4 integrin at its N terminus. Collagen type XVII also interacts with adherens junction proteins, actinin 1 and 4, and delta-catenin to regulate keratinocyte cell polarity. Proteolytic shedding of its ectodomain has implications in wound healing and disease (44, 75, 114).

### Perlecan

Perlecan, encoded by the *HSPG2* gene, is a major component of all BMs and PCMs of chondrocytes (1, 33, 55, 108, 115, 144). The monomeric (~467 kDa) core protein is posttranslationally modified with glycosaminoglycan side chains at its N-terminal domain I. These can be three heparan sulfate side chains in most tissues, or one can be substituted with chondroitin sulfate in intervertebral discs, tendon, and ligaments (34) or a heparan sulfate/chondroitin sulfate/keratan sulfate hybrid in cultured cells (84). The heparan sulfate chains interact with fibroblast growth factor 2 (FGF2) (whereas chondroitin sulfate on the core protein tempers this interaction) to regulate chondrocyte proliferation in growth plates (139).

The five domains of the modular core protein of perlecan have multiple binding partners and functions (for reviews, see 53, 55, 99). Domain I interacts with laminin, fibronectin, and collagen type IV in BMs and with collagen types VI and XI, fibrillin 1, and proline/arginine-rich end leucine-rich-repeat protein in PCMs, where it regulates mechanosensory signals. The cysteine-rich and disulfide-bonded domain II is similar to members of the low-density lipoprotein receptor family, supports low-density lipoprotein retention in the arterial subendothelium, and is implicated in atherosclerosis. Domain III, resembling the short arm of laminin α chains, interacts with FGF7, FGF18, platelet-derived growth factor, von Willebrand–related protein, collagen type VI, and tropoelastin and is implicated in mechanosensory signals in the PCM. Domain IV has multiple disulfide-bonded immunoglobulin-like motifs; interacts with collagen type IV, fibronectin, and entactin/nidogen 1; and is implicated in ECM stabilization. Domain V resembles the tail end of laminin α chains and interacts with entactin/nidogen 1, fibulin 2, ECM1, and collagen type VI. Recombinant domain V (106), termed endorepellin, blocks endothelial cell migration and is itself antiangiogenic through its regulation of phosphotyrosine kinases in an α2β1 integrin–dependent manner (116).

Perlecan serves primarily as a cell signal regulator rather than as a structural component of ECMs. Its functional deficiencies appear to impact cell–PCM interactions of mesenchymal cells in chondrodysplasias and keratoconus, as discussed below (see the section titled Chondrodysplasias: Schwartz–Jampel Syndrome Type 1 and Dyssegmental Dysplasia, Silverman–Handmaker Type and the section titled Keratoconus and Keratocytes).

## BASEMENT MEMBRANE PATHOLOGIES

### Epidermolysis Bullosa

The skin is one of the larger tissues and has the fundamental function of protecting us from external assaults. It is a structured barrier that needs to be flexible and resistant. These characteristics are mediated by tight interactions between keratinocytes and the underlying derma, involving integrins, laminins, and collagens. Accordingly, genetic defects in these proteins cause severe skin pathologies characterized by skin fragility, blistering, and continuous erosion that have been identified as a heterogenous group of rare Mendelian disorders termed EB (60, 61, 151). The junctional type, JEB, is a severe form caused by mutations in *LAMA3*, *LAMB3*, *LAMC2* (encoding subunits of Lm332), *COL17A1* (24) (listed in Table 1), and the integrin genes *ITGA6*, *ITGA3*, and *ITGB4* (encoding the α6, α3, and β4 integrin subunits, respectively) (59). Mutations leading to a complete absence of laminin chains cause the Herlitz form, which is lethal by 6–24 months after birth, while the presence of 5–10% of the proteins causes a milder, nonlethal phenotype. Detailed genotype–phenotype correlations are discussed in References 24 and 59. In JEB, the lamina lucida of the cutaneous BM zone is affected in the skin at sites exposed to friction, trauma, and heat, as well as some internal mucosae. The ocular surface shows variable degrees of corneal erosion, scarring, and vision loss (20, 24, 77).

DEB, the dystrophic forms of EB, is due to mutations in the *COL7A1* gene (encoding collagen type VII) inherited either recessively (RDEB, the most severe form) or dominantly (DDEB). Tissue separation occurs in the anchoring filament and interstitial collagen adhesion zone below the dermal BM but may also affect joints and internal mucosae (61). Milia and pseudosyndactyly are associated with DEB, and life expectancy is significantly reduced due to increased risks of carcinoma development. Corneal blisters and erosions are estimated to occur in 35–74% of patients, scarring in 24–41%, and vision loss in 3–64% (20, 43). The severe forms of RDEB are due to premature termination codons in both alleles that result from nonsense, frameshift, or exon-skipping mutations and total ablation of collagen type VII. Milder phenotypes result from premature termination codons in combination with a missense mutation, or the presence of missense mutations in both alleles. Several excellent reviews have discussed genotype–phenotype correlations (28, 59, 152). A majority of DDEB cases involve glycine missense substitutions in the collagenous triple-helical domain, but some involve nonglycine missense mutations in the noncollagenous NC2 domain. Approximately 10% of all mutations are clustered in exon 73, which corresponds to the evolutionarily conserved narrow hinge-like interruption between the two collagenous domains, emphasizing its functional importance in anchoring fibrils (28). This site is also the target of gene therapy by AON-mediated exon skipping (see the section titled Gene Therapies for Extracellular Matrix–Related Diseases).

### Alport Syndrome

ATS comprises a group of rare familial kidney diseases associated with sensorineural deafness and ocular abnormalities and constitutes approximately 3% of all chronic kidney disease. The underlying causes are genetic defects in collagen type IV that lead to epithelial cell and BM defects, particularly in the kidney glomeruli (22, 46, 65, 141, 157). X-linked Alport syndrome (ATS1), the most common form (85% of all cases, prevalence 1 in 10,000), is caused by variants in *COL4A5* (7, 70) [Table 1; for an updated list of variants, see the ClinVar database (89, 110)]. ATS1 males have relatively homogeneous severe disease, and heterozygous females show a range of localized pathologies due to random X inactivation of the chromosome carrying the wild-type allele. Autosomal ATS, which is relatively rare (prevalence 1 in 50,000), is due to *COL4A3* and *COL4A4* mutations that follow homozygous recessive, compound heterozygous, rare dominant, and possibly digenic modes of inheritance (80). Phenotypically, ATS as a whole is heterogeneous, displaying hematuria, proteinuria, glomerular basement membrane (GBM) thinning, localized lamination, focal segmental glomerulosclerosis, and end-stage kidney disease. Diagnosis is based on glomeruli biopsy ultrastructure, clinical criteria, family history, and genetic testing, with a strong emphasis on identifying individuals who would benefit from early diagnosis and interventions to delay or prevent end-stage kidney disease (81, 157). Whole-genome sequencing and whole-exome sequencing are identifying rare variants that will further improve genetic testing and genotype–phenotype correlations (52). Thus far, according to the ClinVar database (89, 110), 405 of 997 variants in *COL4A3* and 526 of 1,105 variants in *COL4A4* have been reported in confirmed autosomal ATS cases, while 1,075 of 1,801 *COL4A5* variants have been reported in ATS1 cases.

A body of work on collagen type IV, renal cell, and ECM biology provides a greater understanding of pathogenic mechanisms in chronic kidney diseases. The kidney glomeruli collect and filter plasma to retain nutrients and proteins and remove urea and excess water. Their functioning is ensured by three types of ECM (17): the epithelium-derived Bowman’s capsule BM, an internal interstitial mesangial ECM, and a thick GBM. The GBM results from the developmental fusion of BMs produced by specialized epithelial podocytes and the endothelial layer at the capillary end. Selective filtration is mediated by the GBM, intercellular spaces or slit diaphragms between podocyte foot processes, and the fenestrated endothelium. The α112 heterotrimer or protomer occupies the developing GBM, the mesangial ECM, and other BMs, while α556 is limited to the Bowman’s capsule BM. After development, the highly cross-linked and structurally more stable α345 network takes over the podocyte-derived BM of the thick adult GBM (56, 104). Genetic changes in any one α chain can disrupt the association and secretion of the functional α345 protomer (15), and the developmental switch to this isoform is disrupted in ATS (79). Mechanistically, the initial pathology in ATS may arise from an α345 protomer–poor, structurally weak GBM that is unable to counteract high capillary blood pressure. In addition, in the α345 protomer–poor GBM, inappropriate close interactions of the α112 protomer with podocytes via integrins and *DDR1* can cause downstream podocyte pathologies in ATS patients (25).

Studies of genotype–phenotype correlations, collagen type IV structures, and mouse models are providing greater insight into ATS pathogenesis. For example, a pathogenic variant that adds eight amino acids within the α3 NC1 domain in an ATS family was proposed to disrupt the interacting surfaces of two α345 protomers and supramolecular protomer functions in the GBM. A knock-in mouse strain carrying this variant displayed similar GBM disease and α345 protomer ultrastructural defects (120, 121). Mice with targeted deletions in *Col4a3*, *Col4a4*, and *Col4a5* and a spontaneous mutation in *Col4a4* harbor ATS pathologies and serve as mouse models for studying disease onset, progression, and therapies (86, 105). Other genetic and environmental factors can also affect podocyte–GBM adhesion, podocyte loss, and breakdown of the glomerular filtration barrier (27) but are not discussed further here. For ATS therapies, direct correction of the genetic defect has not been achieved. Blood pressure–lowering angiotensin-converting enzyme (ACE) inhibition alleviates GBM tissue damage and reduces proteinuria in *Col4a3* knockout mice and in patients; with increased diagnosis, this treatment is now widely used to delay end-stage kidney disease (81, 121).

## PERICELLULAR MATRIX PATHOLOGIES

### Chondrodysplasias: Schwartz–Jampel Syndrome Type 1 and Dyssegmental Dysplasia, Silverman–Handmaker Type

Two rare chondrodysplasias, SJS1 and DDSH, are due to autosomal recessive mutations in the *HSPG2* gene (3, 5, 99, 142). SJS1 presents as a mild to severe myotonia, muscle atrophy, short stature, myopia, pigeon breast, and cartilage dystrophy, with most individuals being heterozygous, except for a few homozygous individuals arising in consanguineous families (112). DDSH is a neonatal, lethal, generalized chondrodysplasia with micromelia and anisospondyly; the endochondral growth plate is short, with disorganized hypertrophic chondrocytes and defective ossification. DDSH was first reported in two sibs of a consanguineous family with a duplication of 89 base pairs in exon 34 of both *HSPG2* alleles, along with a third, unrelated case who was compound heterozygous for point mutations that caused skipping of exon 52 and 73 (5). Immunostaining of DDSH cartilage from these individuals showed poor staining of perlecan in the PCM, while cultured fibroblasts showed little secretion of sulfated proteoglycans, indicating that DDSH is caused by functional null mutations. Immunohistology on muscle tissues of SJS1 patients showed either reduced staining of domains III–V or an absence of domain V and reduced secretion of perlecan by cultured cells (3). Thus, DDSH, which is more severe, results from having little or no functional perlecan, while SJS1 patients have some functional protein. Although much is known about the functions of the modular core protein domains, no clear correlation is evident between domains affected and SJS1 severity, except that domain I may be essential and its disruption causes loss of protein (99, 143).

Very early on, *Hspg2*-null mice indicated perlecan’s central role in chondrodysplasias. *Hspg2*-null mice die around embryonic day 11.5 (4, 26) due to abnormal cephalic development, while those that survive longer show loss of chondrocyte proliferation and endochondral ossification. The *Hspg*2^−/−^ chondrocytes lack the translucent PCM zone seen in wild-type mice, with altered immunohistological staining for collagen types II and X and agrin in the growth plate, indicating a central role for perlecan in the chondrocyte PCM. On the other hand, mutations in *Unc-52*, the *HSPG2* homolog in *Caenorhabditis elegans*, cause paralysis with disorganized body wall muscle and likely disruptions in integrin-mediated adhesion between myofilaments and the BM (127).

### Keratoconus and Keratocytes

Shinde et al. (134) recently detected two *HSPG2* variants in families with keratoconus, where ECM loss and stromal thinning of the cornea lead to loss of vision. Keratocytes are corneal fibroblast-like cells responsible for producing and maintaining the corneal stroma. Much like chondrocytes, keratocytes are embedded in an interstitial collagen-rich tissue where the PCM is functionally important for cellular homeostasis. The variant p.T2436N affects domain IV, which has a major role in PCM stabilization; the variant p.A4328T affects the terminal globular subdomain of domain V and may disrupt cell–integrin adhesion and interactions with vascular endothelial growth factor A. Unlike DDSH and SJS1, however, keratoconus is likely polygenic, where the accumulation of additional genetic defects is responsible for disease penetrance.

## GENE THERAPY STRATEGIES

### Overview

**Figure 3:**
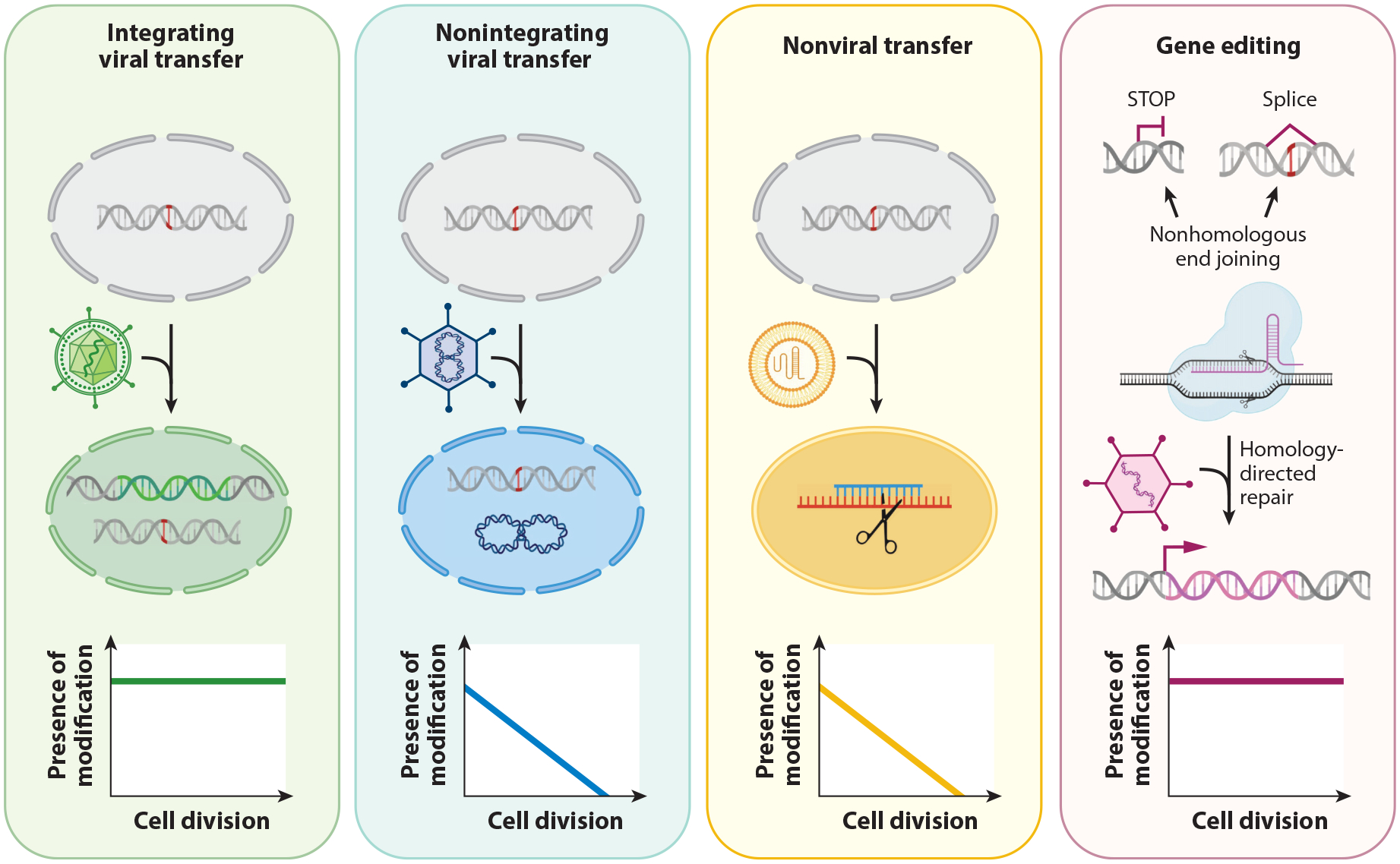
Strategies for introducing genetic material into cells and the persistence of the genetic modifications in dividing cells.

The concept of gene therapy—the introduction of genetic material into a patient to cause functional changes in cells to ameliorate genetic diseases—began in the 1970s (102, 107). However, significant clinical studies on patients did not take off until the 1990s (2, 16). Clinical gene therapy trials to treat rare monogenic diseases are increasing rapidly (74), as are safety considerations. Delivery of genetic material by viral and nonviral means is being developed for therapy, but stable persistence of the genetic material in dividing cells can vary, as summarized in Figure 3. The viral vectors also have varying packaging capacities and the ability to integrate into the genome or remain episomal, and each has its own advantages and limitations, which are summarized in Table 2 (16, 30, 92, 98, 168). For gain-of-function mutations, attempts to silence the expression of the mutated allele are made by viral and nonviral means of delivery.

Nonviral transgene delivery mechanisms include new biomaterials, lipids, nucleic acid–based materials, and nanoparticles. These have the potential to overcome limitations such as host immunogenicity, carcinogenesis, and limited DNA packaging capacity (for reviews, see 58, 66, 87, 162). Major advances have occurred in the nonviral nucleic acid–based field, where a transgene or self-amplifying mRNA introduced into the host expresses the antigen that the host immune system will target. While this approach is being used primarily to treat viral diseases such as coronavirus disease 2019 (COVID-19) (reviewed in 156) and cancer, its broader application is no doubt recognized.

Genome editing, using single guide RNA (sgRNA) and Cas9 endonucleases via plasmid or viral expression vectors, holds the promise of permanent modifications with high impact for many genetic diseases (35, 128, 136). Guided by the sgRNA, the nuclease complex introduces double-strand breaks at specific sites in the genome. During the repair process, nonhomologous end joining introduces small insertions or deletions that can be utilized to excise a pathogenic mutation in vivo. This approach has made significant gains in Duchenne muscular dystrophy (166). On the other hand, precise gene editing is achievable by homology-directed repair of the double-strand break in the presence of a template DNA to introduce site-specific changes. The frequency of homology-directed repair, which is highly dependent on cell type, is also much lower than that of nonhomologous end joining. Off-target cleavage by Cas9, undesirable editing, and techniques to minimize these issues have been discussed elsewhere (124).

Another major consideration is whether the genetic material is directly introduced in vivo or introduced into isolated cells that are expanded ex vivo and then introduced into an individual. Ex vivo expansion uses patient-derived autologous primary cells from the tissue being targeted. Patient-derived induced pluripotent stem cells (iPSCs) are also used since they can differentiate into a cell type of choice. These efforts are promising and are being standardized as ATMPs for somatic cell gene therapies.

### Gene Therapies for Extracellular Matrix–Related Diseases

A combination of viral vectors and ex vivo culture of patient-derived cells is gaining traction in treatments of ECM-related diseases. In *LAMB3* JEB patients, autologous primary keratinocytes, transduced with a retroviral vector expressing the wild-type *LAMB3* cDNA, have been cultured ex vivo and grafted back onto patients (8, 67, 100). The corrected skin grafts displayed healthy adhesive properties between the basal epidermal cells and the underlying derma; a multicenter phase 2/3 clinical trial is in progress based on these results (ClinicalTrials.gov identifier NCT05111600) (30). This study demonstrated (*a*) permanent expression of retrovirally delivered *LAMB3* after transgene integration into target cells and (*b*) long-lasting skin reconstitution by targeting keratinocyte stem cells.

Retroviral gene therapy is also being pursued for RDEB patients in two independent phase 1/2 clinical trials (NCT01263379 and NCT02984085) and a phase 3 clinical trial (NCT04227106) (40, 137). Patient-derived keratinocytes transduced with a *COL7A1* cDNA retroviral vector and cultured ex vivo as grafts were reintroduced at wound sites. The treated areas demonstrated correctly assembled anchoring filaments, indicative of incorporation of the normal α1 chain. In the long run, however, the treated sites showed 50% healing, due to either poor expression of the corrected chain or infiltration of noncorrected native epidermal cells. Compared with the *COL7A1* RDEB efforts, the *LAMB3* JEB treatments enjoyed better long-term skin restoration. Part of the underlying reason may be the reduced proliferative capacity of the mutant *LAMB3* keratinocytes, causing the corrected keratinocytes to have a growth advantage and be the dominant cell type in the graft (32). This emphasizes the need to better understand the underlying biology, the cells to target for therapy, and the cells’ ability to self-renew in vivo (reviewed in 31).

Two clinical trials (NCT04213261 and NCT02493816) (94, 97) describe the expression of *COL7A1* cDNA in RDEB-derived fibroblasts using lentiviral constructs. With some differences, both teams reported limited adverse effects, but complete data on the treatment’s efficacy are not yet fully available. A recent review discussed the use of autologous and allogenic dermal fibroblasts for RDEB treatment (132). Two early RDEB studies attempted to use iPSCs to correctly express collagen type VII. In a first proof of concept, iPSCs derived from a mouse model were corrected and differentiated into fibroblasts and then introduced intradermally, where they secreted correctly assembled collagen type VII (158). In the other study, which used patient-derived iPSCs, the *COL7A1* defect was corrected using conventional gene targeting mediated by adeno-associated viral vectors. Correctly targeted iPSC clones differentiated into keratinocytes and grafted onto mice, as a functional assay, were able to produce skin tissues (131).

Herpes simplex virus 1 has been proposed as a vector in a topical cream (KB103) for treatment of RDEB (107), where the episomal expression of *COL7A1* protein in both keratinocytes and fibroblasts underneath the lesion can be palliative (NCT03536143). However, loss of the transgene with cell proliferation and renewal will require repeated treatments. No clinical trial data are available. Another type of cell-based treatment for RDEB involves bone marrow and mesenchymal stem cell transplantation (NCT00881556 and NCT02582775), but graft rejection, efficacy, and safety issues contribute to poor success at this point (38, 56a, 155).

CRISPR-Cas9-mediated genome editing based on nonhomologous end joining is being pursued for dominant negative *LAMB3* JEB and *COL7A1* DDEB to knock out the mutated alleles such that expression from the normal allele would be enough to rescue the phenotypes. Thus, in a DDEB patient carrying a 15-nucleotide deletion in *COL7A1*, the mutated allele was targeted by nonhomologous end joining in patient-derived iPSCs. Selected iPSC-differentiated keratinocytes and fibroblasts showed that only the wild-type allele product was assembled into homotrimeric collagen type VII, indicating appropriate silencing of the mutant allele (135). Genome editing based on homology-directed repair has been used on primary keratinocytes from three RDEB patients with an insertion or a single-nucleotide variant. In these studies, CRISPR-Cas9 ribonucleoprotein and a template DNA were delivered by adeno-associated viral vectors (10). The edited keratinocyte clones showed expression from the corrected allele and demonstrated assembly of healthy skin architecture in skin grafts in immunodeficient mice. Another, slightly modified approach used on JEB patients introduced a stop codon in intron 2 of the endogenous mutated allele and introduced a promoter-less *LAMB3* cDNA flanked by a splice donor and a poly(A) tail (9). The wild-type protein expressed from the promoter-less *LAMB3* transgene was functionally tested in skin grafts in immunodeficient mice. These studies are bringing genome editing closer to clinical applications.

In a similar vein, two preclinical studies reported the use of exon skipping mediated by AONs to correct collagen type VII defects in RDEB and DDEB. It is worth noting that collagen type VII is particularly well suited for AON-mediated exon skipping, as most *COL7A1* exons are in-frame, and small discrete variants cluster in specific exons. One group used the AON-mediated skipping of exon 105 in keratinocytes derived from patients to rescue collagen type VII synthesis and demonstrated collagen assembly in culture and in reconstituted skin grafts of these cells when placed in athymic immunodeficient mice (13). A clinical study achieved AON-mediated skipping of exon 73 (NCT03605069), which may be useful for topical delivery in RDEB and DDEB patients, but no clinical data are available yet (11). Exon skipping is also being developed for ATS1 therapy; when tested in a mouse model of ATS1, it resulted in correct assembly of collagen type IV trimer and increased survival (160).

## CONCLUSIONS

The collagens, glycoproteins, and proteoglycans discussed here are ubiquitous ECM macromolecules. They occupy cell-adjacent niches, contribute to the matrix barrier, and facilitate critical access of growth factors, cytokines, and signaling cues to cells. Genetic defects in these macromolecules have widespread effects on barrier tissues of the skin, cornea, and kidney and on connective tissues such as cartilage. Their fundamental biology should provide some understanding of the phenotypes associated with their genetic defects. Perlecan, for example, is clearly a multifaceted regulator of growth factor signaling, cell differentiation, and early development. It is a major component of the BM and the PCM, but its functional loss impacts primarily the PCM and impairs chondrocyte differentiation in chondrodysplasias. Collagen type IV and the laminins are major BM components. Because laminin polymers are a primary organizer of the BM, their functional loss leads to a widespread failure of thin BM and blistering skin diseases. Collagen type IV polymers may have a larger role in the thicker BMs, such that defects in their encoding genes impact renal GBM functions; some of these impacts are directly due to structural weakening of the ECM, while others are due to their effect on cellular health.

There has been remarkable progress in cell and gene therapy for a handful of these conditions. For example, in JEB and DEB, introduction of the wild-type transgene in autologous cells, ex vivo expansion, and grafting have reached the clinic. Major advances have occurred in ex vivo expansion of patient-derived keratinocytes, as well as in iPSC technologies and biomaterials and scaffold developments. Treatments of rare perlecan-associated chondrodysplasias require varied approaches, including diagnoses, prenatal genetic screening and counseling, and symptomatic and supportive therapies for patients. With increasing progress in gene-editing approaches through CRISPR-Cas9 and various nucleic acid–based treatments, cell and gene therapy will reach a broad spectrum of ECM genetic disorders in the future.

## Figures and Tables

**Table 1:** ECM-encoding genes and associated pathologies

ECM molecule	Gene	Pathology	OMIM	Transmission	Main tissues/systems/organs involved
**Collagens**					
Collagen type I	*COL1A1*	Caffey disease	114000	AD	Bones
		Ehlers–Danlos syndrome types I, VIIa	130000, 130060	AD, AD	Bones, joints, skin, eyes, CNS
		Osteogenesis imperfecta types I, II, III, IV	166200, 166210, 259420, 166220	AD, AD, AD, AD	Bones, skin, eyes, ears, teeth, cardiovascular system, respiratory system, CNS
	*COL1A2*	Combined osteogenesis imperfecta and Ehlers–Danlos syndrome type 2	619120	AD	Bones, joints, skin, eyes, cardiovascular system
		Ehlers–Danlos syndrome type VIIb, V cardiac valvular type	617821, 225320	AD, AR	Skin, joints, cardiovascular system, CNS
		Osteogenesis imperfecta types II, III, IV	166210, 259420, 166220	AD, AD, AD	Bones, skin, eyes, ears, teeth, heart, respiratory system, CNS
Collagen type II	*COL2A1*	Achondrogenesis type II	200610, 609162	AD	Bones, joints
		Avascular necrosis of the femoral head	608805	AD	Bones
		Czech dysplasia	609162	AD	Bones, joints
		Legg–Calvé–Perthes disease	150600	AD	Bones
		Kniest dysplasia	156550	AD	Bones, joints, eyes, respiratory system
		Platyspondylic skeletal dysplasia, Torrance type	151210	AD	Bones, joints
		Osteoarthritis with mild chondrodysplasia	604864	AD	Bones, joints
		Spondyloepimetaphyseal dysplasia, Strudwick type	184250	AD	Bones, eyes
		Spondyloepiphyseal dysplasia congenita; spondyloepiphyseal dysplasia, Stanescu type; spondyloperipheral dysplasia	183900, 616583, 271700	AD, AD, AD	Bones, joints, eyes, ears, respiratory system, CNS
		Stickler syndrome type I, nonsyndromic ocular	108300, 609508	AD, AD	Bones, eyes, ears, heart
		Achondrogenesis type II, hypochondrogenesis	200610	AD	Bones, muscles
Collagen type III	*COL3A1*	Ehlers–Danlos syndrome type IV	130050	AD	Bones, joints, skin, eyes, cardiovascular system, lungs, genitourinary system
		Polymicrogyria with or without vascular-type Ehlers–Danlos syndrome	618343	AR	Bones, joints, skin, eyes, teeth, cardiovascular system, CNS
Collagen type IV	*COL4A1*	Hereditary angiopathy with nephropathy, aneurysms, and muscle cramps	611773	AD	Muscles, skin, eyes, cardiovascular system, genitourinary system, CNS
		Brain small vessel disease with or without ocular anomalies	175780	AD	Eyes, cardiovascular system, blood, CNS
		Intracerebral hemorrhage, stroke	614519		Cardiovascular system
		Pontine microangiopathy, pontine leukoencephalopathy	618564	AD	Cardiovascular system, CNS
	*COL4A2*	Brain small vessel disease type 2	614483	AD	CNS
		Intracerebral hemorrhage, stroke	614519		Cardiovascular system
	*COL4A3*	Alport syndrome types 2, 3	203780, 104200	AR, AD	Eyes, ears, cardiovascular system, genitourinary system
		Hematuria, familial benign	141200	AD	Genitourinary system
	*COL4A4*	Alport syndrome type 2	203780	AR	Eyes, ears, cardiovascular system, genitourinary system
		Familial benign hematuria	141200	AD	Genitourinary system
	*COL4A5*	Alport syndrome type 1	301050	XLD	Eyes, ears, cardiovascular system, genitourinary system
	*COL4A6*	Leiomyomatosis/Alport syndrome complex	308940	XLD	Eyes, ears, respiratory system, gastrointestinal system, genitourinary system
		X-linked deafness type 6	300914	XLD	Ears
Collagen type V	*COL5A1*	Ehlers–Danlos syndrome type I	130000	AD	Bones, joints, skin, eyes, CNS
	*COL5A2*	Ehlers–Danlos syndrome type II	130010	AD	Bones, joints, skin
Collagen type VI	*COL6A1*	Bethlem myopathy type 1	158810	AD or AR	Bones, muscles, respiratory system
		Ullrich congenital muscular dystrophy type 1	254090	AD or AR	Bones, muscles, skin, respiratory system, PNS
	*COL6A2*	Bethlem myopathy type 1	158810	AD or AR	Bones, muscles, respiratory system
		Congenital myosclerosis	255600	AR	Bones, muscles, respiratory system
		Ullrich congenital muscular dystrophy type 1	254090	AD or AR	Bones, muscles, skin, respiratory system, PNS
	*COL6A3*	Bethlem myopathy type 1	158810	AD or AR	Bones, muscles, respiratory system
		Ullrich congenital muscular dystrophy type 1	254090	AD or AR	Bones, muscles, skin, respiratory system, PNS
		Dystonia type 27	616411	AR	Mouth, respiratory system, CNS
Collagen type VII	*COL7A1*	Dystrophic epidermolysis bullosa	226600	AR or AD	Joints, skin, eyes, gastrointestinal system
		Nonsyndromic congenital nail disorder type 8	607523	AD	Nails
Collagen type VIII	*COL8A2*	Corneal dystrophy, Fuchs endothelial type 1, posterior polymorphous type 2	136800, 609140	AD, AD	Eyes
Collagen type IX	*COL9A1*	Multiple epiphyseal dysplasia type 6	614135	AD	Bones, joints
		Stickler syndrome type IV	614134	AR	Bones, eyes, ears
	*COL9A2*	Multiple epiphyseal dysplasia type 2	600204	AD	Bones, joints
		Stickler syndrome type V	614284	AR	Eyes, ears
	*COL9A3*	Multiple epiphyseal dysplasia type 3 with or without myopathy	600969	AD	Bones, joints, muscles
		Lumbar or intervertebral disc disease	603932		Intervertebral discs
Collagen type X	*COL10A1*	Metaphyseal chondrodysplasia, Schmid type	120110	AD	Bones
Collagen type XI	*COL11A1*	AD deafness type 37	618533	AD	Ears
		Fibrochondrogenesis 1	228520	AR	Bones, joints, cardiovascular system
		Lumbar disc herniation	603932		Intervertebral discs
		Marshall syndrome	154780	AD	Bones, eyes, ears
		Stickler syndrome type II	604841	AD	Bones, eyes, ears
	*COL11A2*	AD deafness type 13	601868	AD	Ears
		AR deafness type 53	609706	AR	Ears
		Fibrochondrogenesis type 2	614524	AD or AR	Bones, respiratory system
		AD otospondylomegaepiphyseal dysplasia	184840	AD	Bones, joints, ears
		AR otospondylomegaepiphyseal dysplasia	215150	AR	Bones, joints, ears, respiratory system
Collagen type XII	*COL12A1*	Bethlem myopathy type 2	616471	AR	Bones, joints, muscles, CNS
		Ullrich congenital muscular dystrophy type 2	616470	AR	Bones, muscles, skin, respiratory system, gastrointestinal system, PNS
Collagen type XIII	*COL13A1*	Congenital myasthenic syndrome type 19	616720	AR	Bones, joints, muscles, respiratory system, gastrointestinal system, CNS
Collagen type XIV	*COL14A1*	Punctate palmoplantar keratoderma type IB	614936	AD	Skin
Collagen type XVII	*COL17A1*	Junctional epidermolysis bullosa	226650	AR	Skin
Collagen type XVIII	*COL18A1*	Glaucoma, primary closed angle	618880	AD	Eyes
		Knobloch syndrome type I	267750	AR	Bones, eyes, genitourinary system, CNS
Collagen type XXV	*COL25A1*	Congenital fibrosis of extraocular muscles type 5	616219	AR	Muscles, eyes
Collagen type XXVII	*COL27A1*	Steel syndrome	615155	AR	Bones, ears, CNS
**Proteoglycans**					
Aggrecan	*ACAN*	Short stature and advanced bone age with or without early-onset osteoarthritis and/or osteochondritis dissecans	165800	AD	Bones, joints
		Spondyloepiphyseal dysplasia, Kimberley type	608361	AD	Bones
		Spondyloepimetaphyseal dysplasia, aggrecan type	612813	AR	Bones, respiratory system
Asporin	*ASPN*	Lumbar disc herniation	603932		Intervertebral discs of the lumbar spine
		Osteoarthritis susceptibility type 3	607850		Bones, joints
Biglycan	*BGN*	X-linked spondyloepimetaphyseal dysplasia	300106	XLR	Bones, CNS
		Meester–Loeys syndrome	300989	XL	Bones, skin, cardiovascular system, CNS
Dystroglycan	*DAG*	Muscular dystrophy-dystroglycanopathy types A9, C9	616538, 613818	AR, AR	Bones, muscles, eyes, respiratory system, CNS
Decorin	*DCN*	Congenital stromal corneal dystrophy	610048	AD	Eyes
Gliomedin	*GLDN*	Lethal congenital contracture syndrome type 11	617194	AR	Muscles, respiratory system, CNS
Glypican	*GPC3*	Simpson–Golabi–Behmel syndrome type 1	312870	XLR	Bones, cardiovascular system, respiratory system, gastrointestinal system, genitourinary system, CNS
	*GPC4*	Keipert syndrome	301026	XLR	Bones, ears, CNS
	*GPC6*	Omodysplasia type 1	258315	AR	Bones, skin, cardiovascular system, genitourinary system
Perlecan	*HSPG2*	Dyssegmental dysplasia, Silverman–Handmaker type	224410	AR	Bones, genitourinary system
		Schwartz–Jampel syndrome type 1	255800	AR	Bones, muscles, eyes, genitourinary system, CNS
Interphotoreceptor matrix proteoglycan 12	*IMPG1*	Vitelliform macular dystrophy type 4	616151	AD	Eyes
Interphotoreceptor matrix proteoglycan 2	*IMPG2*	Vitelliform macular dystrophy type 5	616152	AD	Eyes
		Retinitis pigmentosa type 56	613581	AR	Eyes
Keratocan	*KERA*	Cornea plana type 2	217300	AR	Eyes
Nyctalopin	*NYX*	X-linked congenital stationary night blindness type 1A	310500	XLR	Eyes
Proteoglycan 4	*PRG4*	Camptodactyly–arthropathy–coxa vara–pericarditis syndrome	208250	AR	Bones, joints, cardiovascular system
Versican	*VCAN*	Wagner vitreoretinopathy syndrome type 1	143200	AD	Eyes
**ECM glycoproteins and proteins**					
Adiponectin	*ADIPOQ*	Adiponectin deficiency	612556	AD	Endocrine system
AE-binding protein 1	*AEBP1*	Classic-like Ehlers–Danlos syndrome type 2	618000	AR	Bones, joints, muscles, skin, cardiovascular system, genitourinary system
Agrin	*AGRN*	Congenital myasthenic syndrome type 8	615120	AR	Muscles, respiratory system
Ameloblastin enamel matrix protein	*AMBN*	Amelogenesis imperfecta type IF	616270	AR	Teeth
Amelogin	*AMELX*	Amelogenesis imperfecta type 1E	301200	XLD	Teeth
Anosmin	*ANOS1*	Hypogonadotropic hypogonadism 1 with or without anosmia	308700	XLR	Olfactory system, genitourinary system, endocrine system, CNS
Bone morphogenetic protein–binding endothelial regulator protein	*BMPER*	Diaphanospondylodysostosis	608022	AR	Bones, respiratory system, genitourinary system, CNS
Cartilage intermediate layer protein 1	*CILP1*	Lumbar disc disease	603932		Lumbar discs
Clarin 1	*CLRN1*	Retinitis pigmentosa type 61	614180	AR	Eyes
		Usher syndrome type 3A	276902	AR	Eyes, nose
Cellular communication network factor 6	*CNN6*	Progressive pseudorheumatoid dysplasia	208230	AR	Bones, joints
Cochlin	*COCH*	AD deafness type 9	601369	AD	Ears
		AR deafness type 110	618094	AR	Ears
Acetylcholinesterase	*COLQ*	Congenital myasthenic syndrome type 5	603034	AR	Bones, skin, eyes, ears, cardiovascular system, respiratory system, gastrointestinal system, genitourinary system, endocrine system, CNS
Cartilage oligomeric matrix protein	*COMP*	Multiple epiphyseal dysplasia type 1	132400	AD	Bones, joints
		Carpal tunnel syndrome type 2	619161	AD	Bones, joints, muscles, skin, PNS
		Pseudoachondroplasia	177170	AD	Bones, joints, CNS
Cysteine-rich protein with EGF-like domain 1	*CRELD1*	Atrioventricular septal defect type 2, partial with heterotaxy syndrome	606217	Complex, AD	Cardiovascular system
Collagen triple helix repeat-containing protein 1	*CTHCR1*	Barrett esophagus/esophageal adenocarcinoma	614266		Gastrointestinal system
Dentin matrix acidic phosphoprotein 1	*DMP1*	Hypophosphatemic rickets	241520	AR	Bones, muscles, teeth, ears, genitourinary system
Dentin sialophosphoprotein	*DSPP*	AD deafness type 39 with dentinogenesis	605594	AD	Ears, teeth
		Dentin dysplasia type II	125420	AD	Teeth
		Dentinogenesis imperfecta, Shields types II, III	125490, 125500	AD, AD	Teeth
Extracellular matrix protein 1	*ECM1*	Lipoid proteinosis of Urbach and Wiethe	247100	AR	Skin, eyes, respiratory system, CNS
EGF-containing fibulin-like extracellular matrix protein	*EFEMP1*	Doyne honeycomb degeneration of retina	126600	AD	Eyes
	*EFEMP2*	AR cutis laxa type IB	614437	AR	Skin, cardiovascular system, respiratory system, genitourinary system, CNS
Elastin	*ELN*	AD cutis laxa type 1	123700	AD	Skin, cardiovascular system, respiratory system, genitourinary system
		Supravalvar aortic stenosis	185500	AD	Cardiovascular system
Endoglin	*ENG*	Hereditary hemorrhagic telangiectasia type 1	187300	AD	Skin, eyes, cardiovascular system, respiratory system, gastrointestinal system, CNS
Eyes shut homolog	*EYS*	Retinitis pigmentosa type 25	602772	AR	Eyes
Fibulin	*FBLN1*	Synpolydactyly type 2	608180	AD	Hands, feet
	*FBLN5*	Cutis laxa type Ia	219100	AR	Bones, joints, skin, cardiovascular system, respiratory system, genitourinary system
		Neuropathy hereditary macular degeneration type 3	608895	AD	Bones, joints, muscles, skin, eyes, CNS
Fibrillin	*FBN1*	Acromicric dysplasia	102370	AD	Bones, skin, vocal cords
		Familial ectopia lentis	129600	AD	Eyes
		Geleophysic dysplasia type 2	614185	AD	Bones, joints, skin, cardiovascular system, respiratory system
		Marfan syndrome, Marfan lipodystrophy syndrome	154700, 616914	AD, AD	Bones, joints, muscles, skin, eyes, cardiovascular system, respiratory system, CNS
		MASS syndrome	604308	AD	Bones, skin, eyes, cardiovascular system, respiratory system
		Stiff skin syndrome	184900	AD	Bones, joints, muscles, skin, PNS
		AD Weill–Marchesani syndrome type 2	608328	AD	Bones, joints, muscles, skin, eyes, cardiovascular system, CNS
	*FBN2*	Congenital contractural arachnodactyly	121050	AD	Bones, joints, muscles, eyes, cardiovascular system, CNS
		Early-onset macular degeneration	616118	AD	Eyes
Fibrinogen	*FGA*	Congenital afibrinogenemia	202400	AR	Cardiovascular system
		Familial visceral amyloidosis	105200	AD	Skin, gastrointestinal system, genitourinary system, endocrine system
		Congenital hypofibrinogenemia/dysfibrinogenemia	616004	AD	Cardiovascular system
	*FGB*	Congenital afibrinogenemia	202400	AR	Cardiovascular system
		Congenital hypofibrinogenemia/dysfibrinogenemia	616004	AD	Cardiovascular system
	*FGG*	Congenital afibrinogenemia	202400	AR	Cardiovascular system
		Congenital hypofibrinogenemia/dysfibrinogenemia	616004	AD	Cardiovascular system
Fibronectin-like domain-containing leucine-rich transmembrane protein 3	*FLRT3*	Hypogonadotropic hypogonadism type 21 with anosmia	615271	AD	Bones, ears, olfactory system, genitourinary system, CNS
Fibronectin	*FN1*	Glomerulopathy with fibronectin deposits type 2	601894	AD	Cardiovascular system, genitourinary system
		Spondylometaphyseal dysplasia, corner fracture type	184255	AD	Bones, joints
Fraser extracellular matrix complex subunit 1	*FRAS1*	Fraser syndrome type 1	219000	AR	Bones, eyes, ears, respiratory system, endocrine system, genitourinary system, CNS
Fras1-related extracellular matrix protein	*FREM1*	Bifid nose with or without anorectal and renal anomalies	608980	AR	Bones, joints, gastrointestinal system, genitourinary system
		Manitoba oculotrichoanal syndrome	248450	AR	Bones, joints, eyes, gastrointestinal system, genitourinary system
		Trigonocephaly type 2	614485	AD	Bones
	*FREM2*	Fraser syndrome type 2	617666	AR	Bones, genitourinary system
		Unilateral or bilateral cryptophthalmos, isolated	123570	AR	Eyes
Gliomedin	*GLDN*	Lethal congenital contracture syndrome type 11	617194	AR	Bones, joints, respiratory system, PNS
Hemicitin	*HMCN1*	Age-related macular degeneration type 1	603075	AD	Eyes
Insulin-like growth factor–binding protein acid-labile subunit	*IGFALS*	Deficiency of acid-labile subunit	615961	AR	Bones, endocrine system
Insulin-like growth factor–binding protein 7	*IGFBP7*	Retinal arterial macroaneurysm with supravalvular pulmonic stenosis	614224	AR	Eyes, cardiovascular system
Laminin	*LAMA1*	Poretti–Boltshauser syndrome	615960	AR	Muscles, eyes, CNS
	*LAMA2*	Congenital merosin-deficient or partially deficient muscular dystrophy	607855	AR	Bones, muscles, eyes, respiratory system, CNS
		AR limb-girdle muscular dystrophy type 23	618138	AR	Muscles, CNS, PNS
	*LAMA3*	Junctional epidermolysis bullosa	245660, 226700	AR	Skin
		Laryngo-onycho-cutaneous syndrome	245660	AR	Skin, eyes, teeth, vocal cords, respiratory system
	*LAMA4*	Dilated cardiomyopathy type 1JJ	615235	AD	Cardiovascular system
	*LAMB1*	Lissencephaly type 5	615191	AR	Eyes, ears, CNS
	*LAMB2*	Nephrotic syndrome type 5 with or without ocular abnormalities	614199	AR or AD	Eyes, genitourinary system
		Pierson syndrome	609049	AR	Muscles, eyes, genitourinary system, CNS
	*LAMB3*	Amelogenesis imperfecta type IA	104530	AD	Teeth
		Junctional epidermolysis bullosa	226700, 226651	AD	Skin
	*LAMC2*	Junctional epidermolysis bullosa	226700, 226650	AD	Skin
	*LAMC3*	Occipital cortical malformations	614115	AR	Eyes, CNS
Leucine-rich gene glioma inactivated	*LGI1*	Familial temporal lobe epilepsy type 1	600512	AD	Ears, CNS
	*LGI4*	Neurogenic arthrogryposis multiplex congenita type 1 with myelin defect	617468	AR	Bones, joints, muscles, eyes, respiratory system, CNS, PNS
Latent transforming growth factor–binding protein	*LTBP2*	Primary congenital glaucoma type 3D	613086	AD or AR	Eyes
		Microspherophakia and/or megalocornea with ectopia lentis and with or without secondary glaucoma	251750	AR	Bones, eyes
		Weill–Marchesani syndrome type 3	614819	AR	Bones, joints, eyes, cardiovascular system
	*LTBP3*	Dental anomalies and short stature	601216	AR	Bones, joints, teeth, cardiovascular system
		Geleophysic dysplasia 3	617809	AD	Bones, joints, muscles, cardiovascular system, respiratory system, liver
	*LTBP4*	AR cutis laxa type IC	613177	AR	Bones, joints, muscles, skin, cardiovascular system, respiratory system, genitourinary system
Matrillin	*MATN3*	Multiple epiphyseal dysplasia type 5	607078	AD	Knee and hip pain, skeletal anomalies, irregular epiphyses and metaphyses
		Osteoarthritis susceptibility type 2	140600		Bones, joints
		Spondyloepimetaphyseal dysplasia, Borochowitz–Cormier–Daire type	608728	AR	Bones, joints
Microfibrillar-associated protein 5	*MFAP5*	Familial thoracic aortic aneurysm type 9	616166	AD	Bones, cardiovascular system
Matrix gamma carboxiglutamic acid protein	*MGP*	Keutel syndrome	245150	AR	Bones, joints, skin, ears, cardiovascular system, respiratory system, CNS
Neuron-derived neurotrophic factor	*NDNF*	Hypogonadotropic hypogonadism type 25 with anosmia	618841	AD	Bones, nose, genitourinary system, endocrine system
Netrin	*NTN1*	Mirror movements type 4	618264	AD	Gastrointestinal system, CNS
Netrin G2	*NTNG2*	Neurodevelopmental disorder with behavioral abnormalities, absent speech, and hypotonia	618718	AR	Bones, joints, muscles, eyes, gastrointestinal system, CNS
Otogelin	*OTOG*	AR deafness type 18B	614945	AR	Ears
Peroxidasin	*PXDN*	Anterior segment dysgenesis type 7 with sclerocornea	269400	AR	Eyes
Reelin	*RELN*	Familial temporal lobe epilepsy type 7	616436	AD	CNS
		Lissencephaly type 2 (Norman–Roberts type)	257320	AR	CNS
R-spondin	*RSPO1*	Palmoplantar hyperkeratosis and true hermaphroditism or with squamous cell carcinoma of skin and sex reversal	610644	AR	Skin, teeth, genitourinary system, endocrine system, neoplasias
	*RSPO2*	Humerofemoral hypoplasia with radiotibial ray deficiency	618022	AR	Bones
		Tetra-amelia syndrome type 2	618021	AR	Bones, cardiovascular system, respiratory system, genitourinary system
	*RSPO4*	Anonychia congenita	206800	AR	Nails
Sparc-related modular calcium-binding protein	*SMOC1*	Microphthalmia with limb anomalies	206920	AR	Bones, joints, eyes, cardiovascular system, CNS
	*SMOC2*	Dentin dysplasia type I with microdontia and misshapen teeth	125400	AR	Teeth
Secreted protein acidic cysteine rich	*SPARC*	Osteogenesis imperfecta type XVII	616507	AR	Bones, joints, muscles, CNS
Sushi repeat-containing protein X-linked 2	*SRPX2*	Rolandic epilepsy, mental retardation, and speech dyspraxia	300643	XLD	CNS
Tectorin	*TECTA*	Deafness type 8/12, 21	601543, 603629	AD, AR	Ears
Transforming growth factor beta induced	*TGFBI*	Corneal dystrophy, Avellino type, epithelial basement membrane type, Groenouw type I, lattice type 1, lattice type IIIa, Reis–Bucklers type, Thiel–Behnke type	607541, 121820, 121900, 122200, 608471, 608470, 602082	AD, AD, AD, AD, AD, AD, AD	Eyes
Thrombospondin	*THBS2*	Lumbar disc herniation	603932		Intervertebral discs
Tenascin C	*TNC*	AD deafness type 56	615629	AD	Ears
Tenascin XB	*TNXB*	Ehlers–Danlos syndrome, tenascin X type	606408	AR	Bones, joints, muscles, skin, cardiovascular system, genitourinary system
		Vesicoureteral reflux type 8	615963	AD	Joints, genitourinary system
Thrombospondin-type laminin G domain and EAR repeats	*TSPEAR*	AR deafness type 98	614861	AR	Ears
		Ectodermal dysplasia type 14, hair/tooth type with or without hypohidrosis	618180	AR	Skin, teeth
Usherin	*USH2A*	Retinitis pigmentosa type 39	613809	AR	Eyes
		Usher syndrome type 2A	276901	AR	Eyes, ears
Von Willebrand factor A domain–related protein 3B	*VWA3B*	AR spinocerebellar ataxia type 22	616948	AR	CNS
Von Willebrand factor	*VWF*	Von Willebrand disease types 1, 2 (subtypes A, B, M, N), 3	193400, 613554, 277480	AD, AD or AR, AR	Skin, cardiovascular system, genitourinary system
Wnt1-inducible signaling pathway protein 3	*WISP3*	Progressive pseudorheumatoid dysplasia	208230	AR	Bones, joints, muscles
Zona pellucida glycoprotein	*ZP1*	Oocyte maturation defect type 1	615774	AR	Genitourinary system
	*ZP2*	Oocyte maturation defect type 6	618353	AR	Genitourinary system
	*ZP3*	Oocyte maturation defect type 3	617712	AD	Genitourinary system

Abbreviations: AD, autosomal dominant; AR, autosomal recessive; CNS, central nervous system; ECM, extracellular matrix; MASS, mitral valve prolapse, aortic root diameter at upper limits of normal for body size, stretch marks of the skin, and skeletal conditions similar to Marfan syndrome; OMIM, Online Mendelian Inheritance in Man; PNS, peripheral nervous system; XLD, X-linked dominant; XLR, X-linked recessive. In the transmission column, a blank cell indicates that the mode of transmission is complex or unknown.

**Table 2:** Gene therapy approaches

Vector type	Packaging size	Cellular tropism	Permanent genome modification	Integration of exogenous DNA into the host genome	Research phase in ECM-related pathologies	Number of clinical trials for monogenic diseases	Clinical applications	Key reference(s)
Retrovirus	8 kb	Dividing	Yes	Yes	Clinical	50–100	JEB, RDEB, hematological disorders	2, 16, 31
Lentivirus	8 kb	Dividing and nondividing	Yes	Yes	Clinical	50–100	RDEB, hematological disorders, immunotherapy	2, 16, 31
Herpesvirus	150 kb	Low dividing potential	No	No	Clinical	<10	RDEB, neurological disorders	2, 31
Adeno-associated virus	5 kb	Dividing and nondividing	Yes	Yes	Preclinical	>100	Retinal degeneration, dystrophy, neuromuscular hemophilia	2, 92
Adenovirus	36 kb	Low dividing potential	No	No	Preclinical	<10	Vaccines, immunotherapy	2
Nonviral transfer	Low efficiency	Dividing and nondividing	No	No	Clinical	10–50	Cancer, vaccines	87, 162
Gene editing	Dependent on the delivery method	Dividing and nondividing	Yes	Yes	Animal model, preclinical, clinical	<10	JEB, DEB, hematological disorders, cancer, muscular dystrophy	9, 10, 13

Information compiled from ClinicalTrials.gov (111), the European Union Drug Regulating Authorities Clinical Trials (EudraCT) database (41), and the Gene Therapy Clinical TrialsWorldwide database (74). Abbreviations: DEB, dystrophic epidermolysis bullosa; ECM, extracellular matrix; JEB, junctional epidermolysis bullosa; RDEB, recessive dystrophic epidermolysis bullosa.
